# Anterior Capsule of the Lens: Comparison of Morphological Properties and Apoptosis Induction following FLACS and Standard Phacoemulsification Surgery

**DOI:** 10.1155/2018/7242837

**Published:** 2018-01-11

**Authors:** Alessandra Pisciotta, Michele De Maria, Tommaso Verdina, Elisa Fornasari, Anto de Pol, Gian Maria Cavallini

**Affiliations:** ^1^Institute of Ophthalmology, University of Modena and Reggio Emilia, Modena, Italy; ^2^Department of Surgery, Medicine, Dentistry and Morphological Sciences, University of Modena and Reggio Emilia, Modena, Italy; ^3^Clinical and Experimental Medicine PhD Program, University of Modena and Reggio Emilia, Modena, Italy

## Abstract

**Purpose:**

Comparative evaluation of morphological features of anterior capsules and apoptosis induction in epithelial cells after femtosecond laser-assisted cataract surgery (FLACS) and standard phacoemulsification surgery.

**Methods:**

Group 1: 30 FLACS anterior capsulotomies and Group 2: 30 manual anterior continuous curvilinear capsulorhexes. All patients were operated on by the same experienced surgeon. Morphological features of the anterior capsules and apoptosis induction in epithelial cells were evaluated.

**Results:**

All patients revealed a significant mean best-corrected visual acuity (BCVA) improvement 3 months after surgery, and no major intraoperative nor postoperative complications occurred. The capsular epithelium appeared to be preserved in both groups. Scanning electron microscopy analysis revealed irregular saw-tooth shaped edges in capsules from Group 1 whereas capsules from Group 2 showed regular and smooth edges. A statistically significant higher expression of the downstream apoptotic effector cleaved caspase 3 was observed in Group 1.

**Conclusions:**

The saw-tooth appearance was likely due to the progressive sequence of laser pulses on the capsule. The low energy/high frequency properties of the laser pulse, combined with an overlapped pulse pattern, resulted in highly continuous morphology of capsule edges. The higher apoptosis induction in FLACS group might be due to photodisruption-dependent plasma generation and formation of cavitation bubbles.

## 1. Introduction

Cataract surgery is the most performed surgical intervention worldwide, with an increasing number of surgical procedures up to 24 million per year, according to WHO estimations [1].

For several years, the phacoemulsification technique has been the standard method for cataract extraction and the continuous technical improvements such as the clear corneal microincisions, the use of foldable intraocular lenses, and the application of lower ultrasound energy for the fragmentation of the lens have made cataract surgery efficient and safe. The bimanual microincision cataract surgery (B-MICS) is a minimally invasive variant of the traditional coaxial phacoemulsification, which, allowing clear corneal microincisions of 1.4 mm, uses a sleeveless phaco tip with a separated irrigating chopper that grants a higher stability of the anterior chamber and the possibility of using both right and left hands during the surgical procedure [2, 3].

The latest update in cataract surgery was the introduction of femtosecond laser technology. Starting from femtosecond lasers firstly introduced for corneal surgery with the aim of producing corneal flaps for refractive surgery, most of the newest generation femtosecond lasers operate in cataract surgery at a repetition rate up to 160 kHz combined with an energy range of maximum 10–15 *μ*J [4].

Based on the photodisruption principles, such types of lasers allow us to optimally standardize some of the most difficult parts of the procedure, including capsulotomy, nuclear fragmentation, and corneal incisions, besides providing more accurate and reproducible results, when compared to manual approaches [4].

Until the advent of femtosecond laser technology, manual continuous curvilinear capsulorhexis (mCCC) has been the standard method for the opening of anterior capsule in cataract surgery or refractive lens replacement. Femtosecond laser assisted cataract surgery (FLACS) has now revolutionized this important surgical step, creating predictable and curvilinear capsulotomies which result in improved capsule-intraocular lens (IOL) overlap and better IOL centration and position than with the manual technique. In particular, previous findings demonstrated that FLACS capsulotomies have a repeatable and precise size, are centered, and, consequently, provide better IOL centration and reduced probability of IOL tilt [5–9].

Several studies have examined the morphology of the edges and the smoothness of FLACS capsulotomies, which are created by specific laser wavelength, pulse energy, pulse duration, pulse pattern, repetition rate, and spot size [4]. Preliminary analyses of the capsules showed a postage-stamp aspect with a significant roughness, likely caused by several factors, including the laser energy used to create the capsulotomies [10].

Moreover, posterior capsule opacification (PCO) is one of the most recurring long-term complications of cataract surgery. Many studies have been conducted so far in order to define which factors may influence the development of PCO, including the IOL design, the administration of therapeutic agents, the surgical technique itself, and other surgical procedures to clean the posterior capsule [11, 12].

As reported in the literature, lens capsule is exposed to mechanical or photodisruptive damage, when either capsulorhexis or capsulotomy is performed, with consequent death of the epithelial cells. The hypothesis is that femtosecond laser might reasonably induce apoptosis to a higher extent.

The aim of this study was to evaluate morphological features of the human anterior lens capsule, even at the ultrastructural level, following cataract surgery performed through manual technique and FLACS, respectively. Furthermore, we investigated whether apoptosis was induced in monolayered epithelium beneath the anterior capsule, following the two surgical approaches.

## 2. Materials and Methods

The experimental study was approved by the Human Research Ethics Committee of the University of Modena and Reggio Emilia (Modena, Italy) and was conducted in compliance with the Declaration of Helsinki at the Institute of Ophthalmology, University of Modena and Reggio Emilia. Patients with cataract LOCS III grade 2 or 3 and a correct distance visual acuity of 5/10 or worse were included in the study. Patients were excluded in case of ocular trauma, pseudoexfoliation syndrome, or other ocular comorbidities. The study was performed after written consent of the patients undergoing either traditional manual cataract extraction (B-MICS) or femtosecond laser assisted cataract surgery by using the femtosecond laser Ziemer LDV Z8. All the surgical procedures were performed by the same experienced surgeon (G.M.C.).

### 2.1. Surgical Techniques

A consistent mydriasis was obtained with instillation of 10% phenylephrine and 1% cyclopentolate and a locoregional anaesthesia with peribulbar block was performed (1.5 mL of lidocaine 2% and 1.5 mL of bupivacaine 0.5%).

#### 2.1.1. FSL-Assisted Technique

We carefully applied a disposable suction ring to the eye, centered over the limbus. The suction ring was filled with balanced salt solution (BSS) in order to create a liquid interface, and the mobile arm of the laser system was docked over the corneal apex. The ocular structures were shown by the integrated optical coherence tomography (OCT) system and treatment parameters were determined in a customized manner by using the laser platform settings wizard. The energy and frequency laser pulse settings were 900 mW, 1 MHz for capsulotomy; 950–1000 mW, 2 MHz for phacofragmentation; 1200–1300 mW, 2 MHz for corneal incisions. Laser treatment started with lens fragmentation with an eight-piece pie-cut pattern, followed by an anterior capsulotomy of 5.2 mm diameter. Finally, the laser performed two 1.4 mm corneal microincisions at 10 o'clock and 2 o'clock, respectively. At the end, the suction ring was removed from the eye surface to proceed with the phaco procedure. The surgeon checked the patency of the CCIs using a smooth spatula, first with a perpendicular inclination to the limbus inclination and then with gentle movements along the incision, in order to open it completely from side to side. The anterior chamber was filled with ocular viscoelastic device (OVD) in order to proceed with the removal of the anterior capsule, paying attention to any tissue capsular bridges to avoid capsule fugue.

**Figure 1:**
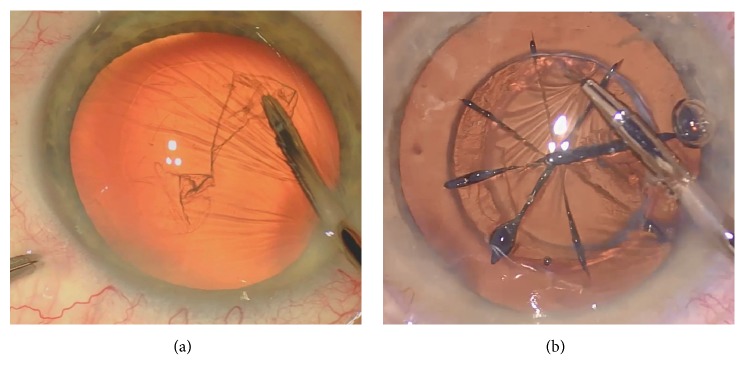
*Anterior lens capsule removal in B-MICS and FLACS surgical techniques*. (a) Manual anterior capsulorhexis with micro-forceps; (b) appearance of the lens after FLACS and anterior capsulotomy removal with micro-forceps.

Hydrodissection and hydrodelineation were performed using a 26-gauge cannula with injection of BSS under the anterior capsule edge, paying attention to the air bubbles movements, in order to avoid any posterior capsular block and ruptures. Bimanual phacoemulsification technique was used to aspirate the nucleus with a 20-gauge 30-degree angled sleeveless probe and a 19-gauge irrigating chopper (Oertli-Instruments AG). Finally, bimanual irrigation/aspiration of the residual cortex was performed using two 21-gauge probes with an oval-shaped section (21-gauge irrigation handpiece smooth, 21-gauge aspiration handpiece rough, Bausch & Lomb) followed by posterior capsule polishing. All intraocular lenses were placed in the capsular bag without complications. Anterior lens capsule removal after either manual anterior capsulorhexis or FSL-assisted anterior capsulotomy is shown in Figure 1.

#### 2.1.2. Standard Technique

The surgeon performed two 1.4 mm biplanar trapezoidal incisions at 10 o'clock and 2 o'clock, respectively, with a precalibrated knife and a manual 5 mm continuous curvilinear capsulorhexis (mCCC). Hydrodissection was performed with 26-gauge cannula and phacoemulsification with a 20-gauge, 30-degree angled sleeveless probe and an irrigating chopper (Bragamele 19-gauge). Phaco-fracture was obtained by the stop-and-chop technique. Irrigation/aspiration was performed with two 21-gauge probes with an oval-shaped section (21-gauge irrigation handpiece smooth Stellaris, 21-gauge aspiration handpiece rough, Bausch & Lomb). In both techniques, IOL was implanted without enlargement of the main incision; the incisions were hydrosutured. BunnyLens AF IOL (Hanita Lenses, Israel) were implanted through 1.4 mm microincision with the wound assisted technique through the ViscoJect™ BIO 1.5 injector. At the end of surgery, OVD was removed and the incisions were hydrated for safety reasons.

Postoperative therapy consisted in tobramycin and dexamethasone eye drops four times a day for 15 days, followed by flurbiprofen eye drops three times a day for further 15 days.

### 2.2. Morphology Evaluation: Phase Contrast, Histological, and Scanning Electron Microscopy Analyses

Anterior lens capsule specimens obtained from either FSL-assisted or standard techniques were immediately washed with phosphate buffer saline (PBS) following surgical extraction. In order to evaluate the morphology and the edges of the capsules obtained following both the cataract surgical techniques, specimens were processed for phase contrast, histological, and scanning electron microscopy analyses. Part of the samples were fixed with 4% paraformaldehyde (PFA) in PBS for 20 minutes at room temperature. Then, samples were washed thrice with PBS and were observed by using a Nikon Eclipse TE2000-U inverted microscope. Representative phase contrast images were acquired with a CCD Hamamatsu ORCA 285 camera. Afterwards, samples were processed for histological analysis, by undergoing dehydration with graded ethanol, clearing and embedding in paraffin. Five *μ*m thick serial sections were cut for each sample and routine haematoxylin/eosin (H&E) staining was performed in order to analyze the morphological details and measure the thickness of the anterior capsules.

Another part of the collected samples was washed, right after surgical procedures, with 0.1 M pH 7.4 Sorenson's Phosphate Buffer (PB) and fixed with 1% glutaraldehyde in 0.1 M pH 7.4 PB. After three washings in PB, anterior lens capsule samples were dehydrated with graded ethanol, until SEM observation was performed. SEM images were acquired with a Nova NanoSEM 450 (FEI) scanning electron microscope (SEM).

The main aim of these analyses was the evaluation of the epithelial cells layer, the presence of fringes in the capsule edges, and the roughness/smoothness of the edges.

### 2.3. Evaluation of Apoptosis in LECs

In order to evaluate whether apoptosis occurred in lens epithelial cells adhering to anterior lens capsule following B-MICS surgical approach and after release of pulse energy by femtosecond laser, samples were processed for confocal immunofluorescence analysis, as previously described [13, 14]. Briefly, capsules fixed with 4% were washed with PBS and underwent saturation with PBS containing 3% bovine serum albumin (BSA) for 30 minutes at room temperature. Then, samples were incubated with a rabbit anti-cleaved caspase 3 primary antibody (Cell Signaling) diluted 1 : 50 in PBS containing 3% BSA, for 1 hour at room temperature. After washing in PBS containing 3% BSA, specimens were incubated with a goat anti-rabbit Alexa546 secondary antibody (Life Technologies), diluted 1 : 200 in PBS containing 3% BSA. Samples were then washed with PBS, stained with 1 mg/ml 4′,6-diamidino-2-phenylindole (DAPI) in PBS for 1 minute, and then mounted with antifading medium. Fluorescent samples were observed by a Nikon A1 confocal laser scanning microscope. The confocal serial sections were processed with ImageJ software to obtain three-dimensional projections and image rendering was performed using Adobe Photoshop Software [15].

### 2.4. Statistical Analysis

Data were evaluated by statistical software (GraphPad Prism Software version 7 Inc., San Diego, CA, USA). Differences between the two experimental groups were analyzed by Student's *t*-test. Data were expressed as mean ± SD. Each experimental evaluation was performed in triplicate. In any case, significance was set at *P* < 0.05.

## 3. Results

### 3.1. Clinical Observations

For Group 1 (FLACS), 30 eyes (15 right eyes and 15 left eyes) of 30 patients (10 males and 20 females) were included in the study; the average age was 74.07 ± 8.48 years. For Group 2 (B-MICS), 30 eyes (13 right eyes and 17 left eyes) of 30 patients (8 males and 22 females) were included in the study; the average age was 75.72 ± 9.16 years. BunnyLens AF IOL was implanted in all eyes. All IOLs were implanted in the bag.

The total surgical time was 17.89 ± 3.80 minutes in Group 1, whereas in Group 2 it was 12.63 ± 2.10 minutes, with a difference resulting to be statistically significant (*P* < 0.05). In Group 1, no incomplete capsulotomy nor capsule buttons were observed; moreover, no intraoperative major complication nor any postoperative complication was recorded in both groups.

Three months after surgery, a mean BCVA improvement of 0.460 ± 0.249 LogMar was observed in Group 1 (*P* < 0.05 versus baseline values). Similarly, in Group 2 a mean BCVA improvement of 0.417 ± 0.150 LogMar (*P* < 0.05 versus baseline values) was observed, at the same evaluation time. No statistically significant differences in the improvement of visual acuity were observed between the two groups.

With regard to astigmatism and corneal pachymetry, we did not detect any statistically significant difference between baseline and postoperative values, with Student's *t*-test either within each group or between the two groups (*P* > 0.05).

Finally, with regard to the endothelial cell count (ECC), in Group 1 at 3-month follow-up we found a mean endothelial cell loss of 162.27 ± 225.11 cells/mm^2^ and of 322.52 ± 339.93 cells/mm^2^ after surgery in Group 2, a reduction that was statistically significant either within each group or between the two groups (*P* < 0.05).

### 3.2. Morphological Analysis: Phase Contrast, Histological, and Scanning Electron Microscopy Analyses

**Figure 2:**
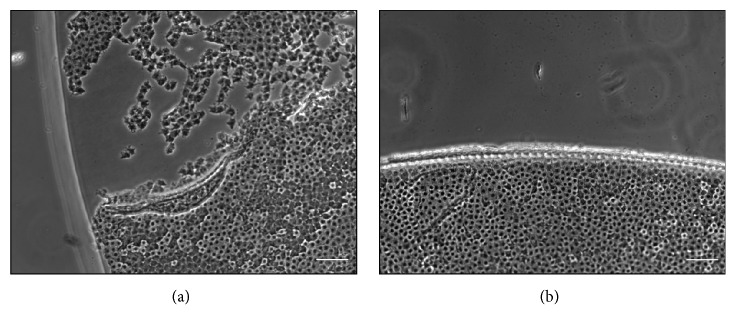
*Histomorphology evaluation of anterior lens capsules*. Phase contrast images showing edges and epithelial layer of anterior lens capsules obtained from B-MICS technique (a) and femtosecond laser assisted cataract surgery (b). Bar: 100 *μ*m.

**Figure 3:**
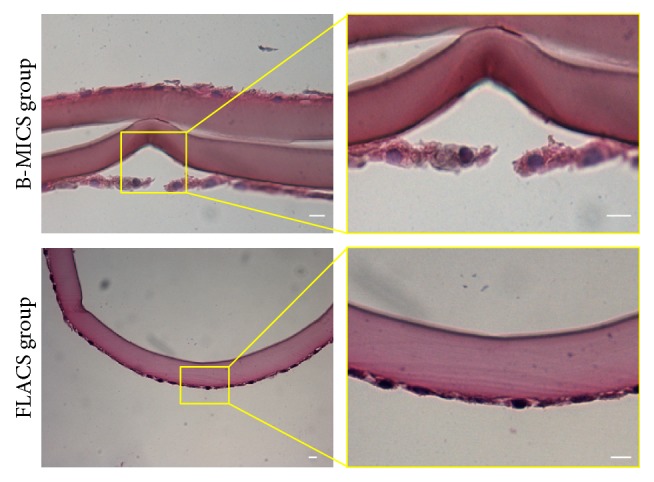
*Histological analysis of anterior lens capsules*. Cross sections of the specimens were stained with haematoxylin and eosin. On the right side, higher magnification images show details of the epithelial layer in specimens obtained following B-MICS and FLACS techniques. Bar: 10 *μ*m.

Phase contrast images showed epithelial cells with well-conserved nuclei and cytoplasm, besides revealing intact cellular borders through most of the epithelial surface, except for the closer proximity of the capsular edges, where epithelial cells were detached from the basement membrane (Figure 2(a)). Edges of the capsules appeared regular and smooth, as reported in Figure 2(a). As far as the capsular appearance in FLACS group is concerned, epithelial cells were mostly adherent to the basement membrane whereas edges showed a less regular, saw-tooth pattern (Figure 2(b)). Histological analysis confirmed that some of the samples from the B-MICS group showed a partial detachment of the epithelial layer from the basement membrane, whereas most of the capsules from the FLACS group demonstrated a continuous adhesion of epithelial cells and basement membrane (Figure 3). Furthermore, measurements of the capsular thickness among the two experimental groups revealed a difference, although without statistical significance. In particular, samples obtained after FLACS technique showed a higher thickness when compared to capsules derived from B-MICS (data not shown).

**Figure 4:**
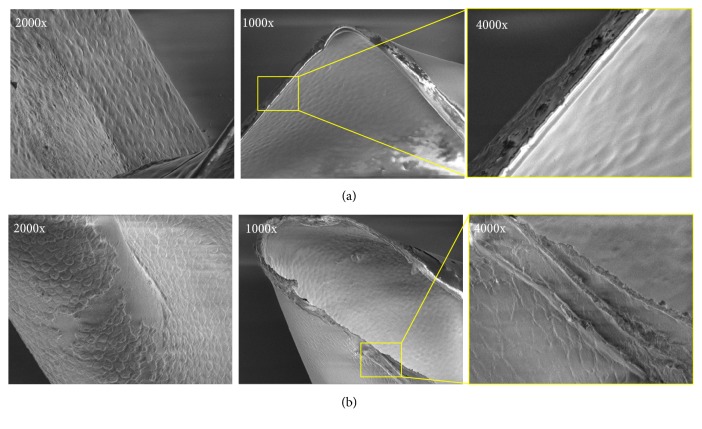
*Scanning electron microscopy analysis*. Representative images of samples obtained with B-MICS (a) and (b) FLACS techniques, showing epithelial layer (left) and edges appearance (right) at different magnifications.

Finally, SEM analysis, as reported in Figure 4, revealed with a higher definition how uniform the adhesion of epithelial cells to the basement membrane was and, most of all, the appearance of capsular edges. In particular, in the B-MICS group, the capsular edges had a regular, smooth, and translucent rim (Figure 4(a)). Otherwise, the margins of capsules obtained through FLACS clearly demonstrated irregular and fringed appearance (Figure 4(b)).

### 3.3. Evaluation of Apoptosis in LECs

**Figure 5:**
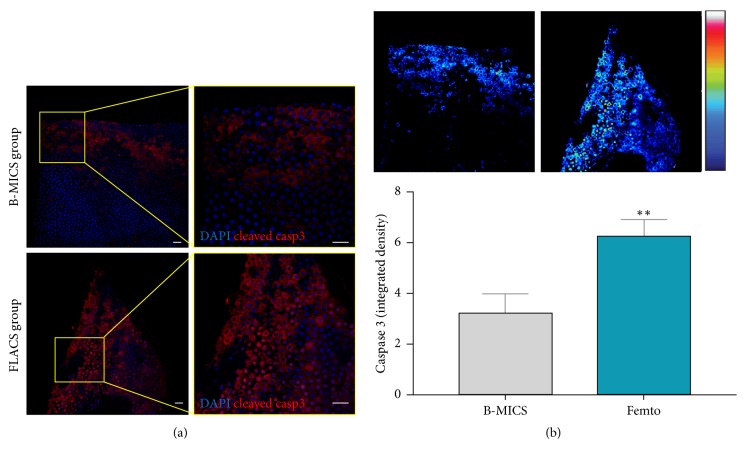
*Evaluation of cell apoptosis in epithelial cells adherent to anterior lens capsules*. (a) Immunofluorescence analysis was performed against cleaved caspase 3 (red), and nuclei were counterstained with DAPI (blue). (b) Pseudocolor images (upper) and densitometric analysis (lower) of cleaved caspase 3 in epithelial cells. Bar: 20 *μ*m.

Confocal immunofluorescence analysis revealed that apoptosis occurred in lens epithelial cells of samples obtained from either the B-MICS group or the FLACS group, as shown in Figure 5(a). While in capsules obtained from the manual surgical technique the presence of cleaved caspase 3 was observed mainly in epithelial cells closer to the capsular edges, samples collected following FLACS technique showed a more spread staining against cleaved caspase 3, from the rim to the core of the epithelial layer. Pseudocolor images of cleaved caspase 3 were obtained as previously described [16] and reported in Figure 5(b). Densitometric analysis carried out on the immunofluorescent signals provided semiquantitative data of the expression of the apoptotic effector cleaved caspase 3, which was statistically significantly higher in the FLACS group, with respect to the B-MICS group (Figure 5(b); ^*∗∗*^*P* < 0.01).

## 4. Discussion

The use of femtosecond lasers constitutes the newest achievement in cataract surgery, starting from their first clinical application in 2008. Due to the increasing necessity to address the expectations of optimum refractive outcome, a lot of new developments have been fulfilled.

The application of femtosecond laser to cataract surgery has allowed improving the safety and effectiveness of the procedure, as well documented in literature [5, 7, 9, 17]. Indeed, previous reports demonstrated the high repeatability and precision in execution of the most difficult phases of cataract surgery, including optimized anterior capsulotomies which produced a continuous sharp-edged cut with good tensile strength [9, 18, 19].

Our study evaluated the morphological features of human anterior capsules obtained with standard phacoemulsification technique and FLACS, respectively. The latter ones were collected following the use of Ziemer LDV Z8, a femtosecond laser that takes advantage of a low energy high frequency system (high pulse repetition rate—up to 10 MHz—combined with a very low energy range of 100–150 nJ) [20] and uses a liquid optic interface to ease the capsulotomy and lens fragmentation phases. These properties allowed obtaining anterior capsule specimens owning a well-preserved histomorphology, with epithelial cells uniformly adherent, when compared to samples obtained after manual technique, as demonstrated by phase contrast and SEM analyses. In accordance with the existing literature, the edges of anterior capsules derived from FLACS were less regular and characterized by a saw-tooth pattern, when compared to the smooth rim produced by manual technique [21, 22]. The pattern observed in the FLACS group is most likely attributable to high density pulse raster and high precision optics of the Femto LDV Z8 laser, which result in multiple overlapping spots that create a continuous clean cut, resembling the one obtained with B-MICS technique [23].

Anterior capsule specimens obtained through FLACS technique also showed a higher thickness, when compared to capsules derived from B-MICS, most likely due to the presence of cortex adherent to the anterior capsule.

Besides histomorphological characteristics, our data showed that apoptosis occurred in epithelial cells of anterior capsules obtained from both surgical techniques. In particular, cleaved caspase 3 expression was significantly higher in samples collected following FLACS, with a positive staining not limited to the cells along the cutting edge but also spread to the inner core of the capsule, thus suggesting a wider induction of cell death. Conversely, in the epithelial layer adhering to the capsule specimens derived from B-MICS procedure, apoptosis was detected only along the cutting rim, as a consequence of mechanical injury induced by the forceps mediated extraction of anterior capsule. This greater apoptotic event affecting the monolayered epithelium beneath the anterior capsule after FLACS is likely due to laser pulse energy, photodisruption-dependent plasma formation, and subsequent creation of cavitation bubbles [4]. Our data are consistent with previous findings from Mayer et al. [24] and, particularly, a spread expression of the apoptotic effector cleaved caspase 3 might represent a positive predicting factor that allows preventing capsular bag opacification, by strongly inhibiting the proliferation of residual epithelial cells which generally correlates to postoperative inflammatory response [25, 26]. From a clinical point of view our study highlighted that, 3 months after surgery, patients from either FLACS or B-MICS groups showed a statistically significant improvement in best-corrected visual acuity (BCVA) values, with respect to the baseline measurements. Noteworthy, as far as endothelial cell count is concerned, a statistically significant decrease in corneal endothelial cell loss was observed in FLACS group, event that is attributable to shorter phacoemulsification times. These data are in accordance with previous findings demonstrating that laser cataract surgery is less traumatic to the corneal endothelium besides offering a tool for surgeons to standardize the critical phases of cataract surgery, through an initial learning curve [27–29].

## 5. Conclusions

The use of femtosecond laser in cataract surgery allows optimizing some of the most difficult steps of the procedure, including capsulotomy, nuclear fragmentation, and corneal incisions, besides providing more accurate and repeatable outcomes. Our findings are in accordance with the existing literature; however further studies will be needed to match the bright sides of manual technique and femtosecond laser, by defining the optimal combination of laser energy, repetition rate, and spot size, in order to obtain better quality capsulotomies with regular cutting edges comparable to those of manual capsulorhexis, and to reduce the inflammatory response in the eye, thus, preventing lens epithelial cell proliferation and capsular bag opacification.

## Abbreviations

FLACS: Femtosecond assisted laser cataract surgery
B-MICS: Bimanual microincision cataract surgery
mCCC: Manual continuous curvilinear capsulorhexis
IOL: Intraocular lens
PCO: Posterior capsule opacification
BSS: Balanced salt solution
OCT: Optical coherence tomography
OVD: Ocular viscoelastic device
PBS: Phosphate buffer saline
PFA: Paraformaldehyde
H&E: Haematoxylin/eosin
SEM: Scanning electron microscope
BSA: Bovine serum albumin
DAPI: 4′,6-Diamidino-2-phenylindole.
